# Cross‐Frequency Couplings Reveal Mice Visual Cortex Selectivity to Grating Orientations

**DOI:** 10.1002/brb3.70360

**Published:** 2025-03-13

**Authors:** Zahra Ebrahimvand, Mohammad Reza Daliri

**Affiliations:** ^1^ Neuroscience & Neuroengineering Research Lab., Biomedical Engineering Department, School of Electrical Engineering Iran University of Science & Technology Tehran Iran

**Keywords:** cross‐frequency coupling, mouse visual cortex, oriented grating, visual perception

## Abstract

**Introduction:**

Oriented grating is usually employed in visual science experiments as a prominent property of neurons in the visual cortices. Previous studies have shown that the study of mouse vision can make a significant contribution to the field of neuroscience research, and also the local field potential (LFP) analysis could contain more information and give us a better view of brain function.

**Methods:**

In this research, cross‐frequency coupling is employed to assess the grating orientation perception in V1 and lateromedial (LM) of 10 mice. The experimental data were collected using chronically implanted multielectrode arrays, involving area V1 recording of five mice and area LM recording of five mice separately, performing a passive visual task. Two criteria known as phase–amplitude coupling (PAC) and amplitude–amplitude coupling (AAC) were exploited to analyze the characteristics of cross‐frequency coupling of LFP signals in the experiment consisting of first‐order and second‐order drifting sinusoidal grating stimuli with different orientations.

**Results:**

It was found that in area LM the correlation between phase of lower than 8 Hz band signal and amplitude of above 100 Hz band signal can be significantly different for orientations and stimulus conditions simultaneously. In area V1, this difference was observed in amplitude correlation between 12 and 30 Hz and more than 70 Hz subbands.

**Conclusions:**

In conclusion, PAC and AAC can be proper features in orientation perception detection. Our results suggest that in both areas, the significant role of high‐band and low‐band oscillations of LFPs discloses the reliability of these bands and generally LFP signals in mice visual perception.

## Graphical abstract

Cross‐frequency coupling is employed to assess the grating perception in V1 and LM of 10 mice, performing a passive visual task. In area LM, phase–amplitude coupling (PAC) between lower than 8 Hz band and above 100 Hz band signals was significantly different for orientations and stimulus conditions simultaneously. In area V1 this difference was observed in amplitude–amplitude coupling (AAC) between 12 and 30 Hz and more than 70 Hz subbands. Our results suggest that in both areas, there is a significant role of local field potential (LFP) signals in mice visual perception.

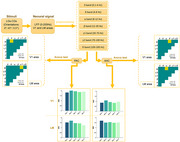

## 1 Introduction

Studies of visual perception have typically relied for decades on species with high visual acuity, such as ferrets, cats, and nonhuman primates. However, the development of functional brain analysis as an essential tool for exploring brain activities led to the mouse being considered as a suitable model system (Huang 2014; Harris and Shepherd 2015). Oriented gratings are useful stimuli for visual science experiments (Baker and Mareschal 2001) and orientation selectivity which is defined as the detection of oriented stimuli, has a considerable value in the perception of the visual world (Priebe 2016; Antinucci et al. 2016). Hubel and Wiesel studied this trait of perception for the first time in cat primary visual cortex (Hubel and Wiesel 1962). Brain distinct rhythms are associated with different spatial scales and thus different cell population sizes, where the correlation between two spatial points in lower rhythms is greater than in higher rhythms (Nunez and Srinivasan 2006; Canolty et al. 2007). It means activity in large spatial regions modulated by lower rhythms and vice versa (Von Stein and Sarnthein 2000). Activity in large spatial regions is often modulated by lower frequency rhythms because these lower frequencies are associated with long temporal windows, allowing for widespread neural coordination and integration over large networks. Conversely, higher frequency rhythms tend to modulate activity over smaller spatial areas, as they operate on shorter time scales and can facilitate rapid, localized processing of information. This functional distinction highlights how different frequency bands serve various roles in neural communication and organization. There are different spatial scales to record the electrical activity of the brain. The local field potential (LFP) recording as synchronized population activity of numerous nerve cells, reflects the simultaneous activity of multiple cortical areas (Destexhe and Bédard 2022). By decomposing neuronal activities into distinct small packages, each of which is in a specific frequency band, the interactions between them are considered a beneficial and important method in neuronal computation, communication, and learning called cross‐frequency coupling (CFC; Schroeder and Lakatos 2009).

In addition to many behavioral studies that have assessed visual perception (Johnson et al. 2020; Masis et al. 2020; Luongo et al. 2023; Schnabel et al. 2018; Caramellino et al. 2021; Reinagel 2015; Tesileanu et al. 2022), several electrocorticographic studies have tried to recognize the relation between brain circuity and visual perception which are summarized in the following. Mesoscale‐level study in higher order mammals (Li et al. 2021) showed CFC functionality in their orientation selectivity in V1 and V4 areas. As the contrast of drifting sinusoidal gratings increases, the spectral responses of neurons in gamma band (compared to baseline activity) exhibited an increase in power, which is different than increases observed in higher or lower frequency bands (Henrie and Shapley 2005; Berens 2008; Berens et al. 2008; Jia et al. 2011). Electrocorticographic recording of V1 during freely natural image viewing has shown that the gamma‐band oscillations play a significant role in perception of natural images (Brunet et al. 2015). Conversely, the binocular rivalry test (Gail et al. 2004) did not support the hypothesis that cortical synchronization at gamma frequencies explains the links of the local parts of a perceived visual object. Furthermore, lower than 30 Hz frequencies resulted in modulations with the perception while these results were not visible in multiunit activity (MUA). Additionally, the power of high‐frequency (> 80 Hz) LFPs recorded from area V1 of macaques (Dubey and Ray 2020), as local origins signals, showed a proper tuning to stimulus orientation, contrast, and spatial frequency. The power of these frequencies has followed a different path compared to the gamma band (Ray and Maunsell 2011; Ray et al. 2008) for varying the stimulus size, suggesting that the two bands have different origins and are separate. These studies reveal the importance of assessing different frequency bands in perception analysis. In this study, we used seven different frequency bands of neuronal activity.

Recording of spiking and LFP activities in area V1 during the presentation of a colored movie to anesthetized macaque monkeys resulted in a strong correlation between high‐frequency LFPs and spikes that suggested they are generated within the same network. On the other hand, very few correlations of low‐frequency LFPs with high‐frequency LFPs and spikes reflected their separating (Belitski et al. 2008). Neural activity and perceptual suppression in monkeys' V1, V2, and V4 areas while doing generalized flash suppression (GFS) explained LFP power appropriate modulation in all three areas while spike‐level scrutiny did not have such comprehensive results (Wilke et al. 2006). Moreover, these results showed that low‐frequency LFP power, compared to high‐frequency LFP or spiking, is more closely related to the representation of stimulus visibility. A model based on the relation between MUA and LFP in all layers of V1 of awake ferrets while freely viewing nature movie clips provided evidence about V1 switching between two distinct states defined by the low and high spectral contents, that is, frequency bands including (delta, alpha) and gamma activities (Sellers et al. 2015). Time investigation of neural firings caused from natural images in area V1 of alert cats showed that the first few tens of milliseconds are dedicated to basic operations of scene analysis (Maldonado and Babul 2007). Because of LFP's high‐frequency band similarity to spike‐level information and also its low‐frequency band key role in visual perception analysis, it was shown that the LFP signal is reliable. Hence, besides the firing rate analysis performed in (Khastkhodaei et al. 2016), we reasoned that the LFP analysis could contain more information and give us a better view of brain function.

In rodent visual perception studies, unlike ferrets (Sellers et al. 2015), learning of oriented grating stimulus recognition resulted in an alteration of visual cortex (V1) power, which augmented in low‐frequency power and reduced in high‐frequency power (Hayden et al. 2021). An increase in contrast of two orthogonal orientations of drifting gratings caused discriminability enhancement in mice area V1 (Long et al. 2015). Assessment of context influence on mouse primary visual cortex neural processing by the oddball paradigm (Hamm et al. 2021) exhibited a reduction of LFP response to the redundant stimulus but it augmented to deviant one, which this augmentation happened by a specific subset of neurons. Interestingly, if prefrontal inputs to V1 were suppressed, contextual selectivity of deviance‐detecting ensembles was reduced. The lowest discriminable coherence thresholds of motion direction in mice and rats sometimes were similar to human levels, on average were two to three times higher than humans, which expressed visual system similarity between rodents and primates (Douglas et al. 2006; Samonds et al. 2018). A discrimination task known as two‐alternative forced‐choice (2AFC) revealed that mice subjects have high‐level performance in discriminating small differences in orientation (Lyamzin et al. 2021). Compared with the dorsal lateral geniculate nucleus (dLGN) (Zhao et al. 2013), current source density analysis on extracellular recordings of anesthetized mice V1 showed neurons selectivity for orientation and spatial frequency, like other species (Niell and Stryker 2008). Despite the temporal contiguity hypothesis, extracellular recordings in rat V1 showed the orientation selectivity stayed the same in increasing and decreasing spatial frequency (Crijns et al. 2019; Crijns 2019). A spike‐level study (Senzai et al. 2019) surveyed the relationship between single‐neuron firing and mesoscopic LFPs in a multilayered configuration of V1 while the mice slept or walked around freely. The results of this study revealed that coherence and spike‐LFP coupling in gamma band identify several physiological layers and sublayers in mice V1 cortices. These studies show that the study of mouse vision can make a significant contribution to the field of neuroscience research, like other species.

In cognitive processing, several types of couplings can be defined based on amplitude, phase, and frequency of the neural signal. Phase synchronization cross‐frequency (Sauseng et al. 2008) has a potential role in the regulation of communication between significantly different rates. Amplitude synchronization (amplitude–amplitude coupling [AAC]), which was correlated with behavior (Shirvalkar et al. 2010), is defined as the correlation of two signal envelopes (Yeh et al. 2016). Phase–amplitude coupling (PAC)—the mostly used metric from CFC family—plays a key role in the various brain functions of primates (Khamechian and Daliri 2020; Zaleshin and Merzhanova 2019) and rodents' hippocampus, and basal ganglia (Tort et al. 2008). PAC has a physiological implication which can be explained as follows: the phase extracted from low‐frequency content indicates local neuronal excitability, whereas the power extracted from high‐frequency content specifies two scenarios: either synaptic activity of neural population or the selective activation of a connected neuronal subnetwork. Investigating the coupling of different brain oscillations during the presentation of visual stimuli provides valuable insights into the timing and coordination of neural activity involved in perception. To the best of our knowledge, no studies have yet applied these methodologies to analyze visual perception in mice utilizing LFP signals recorded from cortical areas. For this study, we measured the PAC and AAC of LFPs in V1 and LM.

## 2 Methods and Materials

### 2.1 Dataset

We used the LFP signals of the dataset recorded by Khastkhodaei et al. (2016). Visual stimuli were two‐dimensional moving sine wave first‐order, luminance‐modulated gratings (LGs) and second‐order, contrast‐modulated gratings (CGs). Each stimulus was shown in eight different orientations, 0°–315° at 45° intervals, with 30 trials for each orientation and in pseudorandom order. Extracellular LFP signals were recorded at sampling frequency of 1.25 kHz from V1 and LM cortices using 32‐channel silicon probes in a four‐shank configuration. Neural data from the aforementioned areas were collected in separate sessions. Each trial was 3 s long, containing 0.5 s prestimulus and 0.5 s poststimulus parts in which a gray screen was shown, and 2 s duration of the grating stimulus showing passively. Our data involve the area V1 recording of five mice and the area LM recording of five mice separately. Comprehensive details of stimuli were explained in Khastkhodaei et al. (2016).

### 2.2 Preprocessing

The primary preprocessing steps (removing noise from the signal, such as electrocardiogram interference, power frequency interference, etc.) had previously been performed by the group that recorded the data (Khastkhodaei et al. 2016). Each session lasted approximately 30 min and was conducted continuously. During these sessions, each grating stimulus was displayed for 2 s, followed by a 1‐s gray screen between stimuli. In total, 18 different stimuli were presented, with each stimulus shown to the subject 30 times.

Before breaking the whole session signal into each trial, we first used a phase‐shift‐free three‐order Butterworth (Crijns et al. 2019; Meneghetti et al. 2021; Hayden et al. 2023) filter to separate the signal into different frequency bands (δ (0.1–4 Hz), θ (4–8 Hz), α (8–12 Hz), β (12–30 Hz), γ1 (30–70 Hz), γ2 (70–100 Hz), and H (100–200 Hz)). After filtering the data from the entire session, we defined each trial as comprising 0.5 s prior to the stimulus presentation, 0.5 s following it, and the 2 s during the display of the grating stimulus. Once the data were segmented into trials, the stimulus display section was normalized relative to the prestimulus period using the *z*‐score method. All analyses were performed on the signal corresponding to the duration of the grating stimulus presentation. Due to the proximity of the channels to each other, we averaged the signals of each octrode (the eight channels on each shank were treated as an octrode) and then broke into trials and normalized to the prestimulus part.

### 2.3 Phase–Amplitude Coupling

CFC of brain fluctuations constructively helps to a better insight into the neuronal mechanisms of perception. To calculate CFC in this study, at first, we determined the Hilbert transform of the signal as below (Jafakesh et al. 2016):

(1) $$\tilde{x}\;\left(n\right)=H\left(x\;\left(n\right)\right)=\left\{\begin{array}{c} \frac{2}{\pi }\sum_{n\;\mathrm{odd}}\frac{x\left(n\right)}{k\;-\;n};\;k\;\mathrm{even}\\ \frac{2}{\pi }\sum_{n\;\mathrm{even}}\frac{x\left(n\right)}{k\;-\;n};\;k\;\mathrm{odd} \end{array}\right.\;$$

The amplitude of the signal *x*(*n*) is defined as follows:

(2) $$A_{x}\;\left(n\right)=\sqrt{\tilde{x}{\left(n\right)}^{2}+x{\left(n\right)}^{2}}\;$$

Also, the instantaneous phase of the signal *x*(*n*) is defined as follows:

(3) $$\varphi_{x}\;\left(n\right)=\;\arctan\left(\frac{\tilde{x}\left(n\right)}{x\left(n\right)}\right)$$

Then for signals *x*(*n*) and *y*(*n*) from different frequency bands, in which *x*(*n*) is from the lower band, PAC is defined as below:

(4) $$\mathrm{PAC}\;=\frac{\left|\frac{1}{N}\sum_{n}p_{y}e^{j\varphi_{x}}\right|}{\sqrt{\sum_{n}p_{y}}}\;$$

where $p_{y}$ is *y* (n) power and square of the amplitude and $\varphi_{x}$ is the phase of x(n) in time step *n*. According to the seven predefined frequency bands, we obtained 21 interband PAC values.

### 2.4 Amplitude–Amplitude Coupling

This kind of coupling is estimated by Pearson correlation between the amplitudes of signals.

(5) $$\mathrm{AAC}\;=\;\mathrm{corr}\;\left(A_{x}\left(n\right),\;\;A_{y}\left(n\right)\right)=\frac{\mathrm{cov}\left(A_{x}\left(n\right),\;\;\;A_{y}\left(n\right)\right)}{\sigma \left(A_{x}\left(n\right)\right).\;\sigma \left(A_{y}\left(n\right)\right)}\;$$

Its value ranges between −1 and 1; where Corr (*x*, *y*) > 0 means that *y* and *x* signals behave identically in a linear fashion. In contrast, Corr (*x*, *y*) < 0 indicates negative linear relation between two signals. Furthermore, Corr (*x*, *y*) = 0 illustrates that there is no linear correlation, hence no coupling between two signals. AAC values for 21 interbands were calculated according to seven predefined frequency bands.

### 2.5 Statistical Analysis

To investigate differences in couplings in response to LGs and CGs and to find the pair bands due to best orientation selectivity, a two‐way analysis of variance (ANOVA) test was conducted under the effects of multiple factors (orientations, stimuli, and trials) for each area.

## 3 Results

**FIGURE 1:**
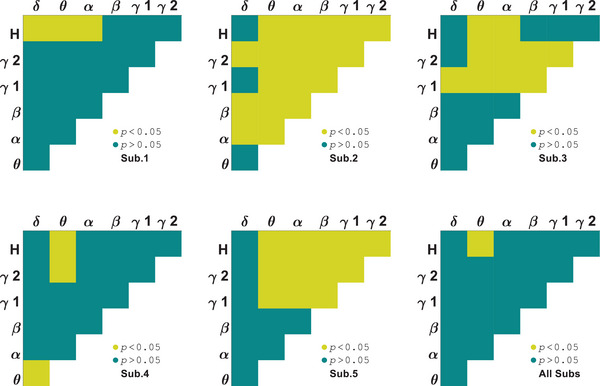
Subbands orientation selectivity of area V1 by analysis of variance (ANOVA) test on phase–amplitude coupling (PAC).

**FIGURE 2:**
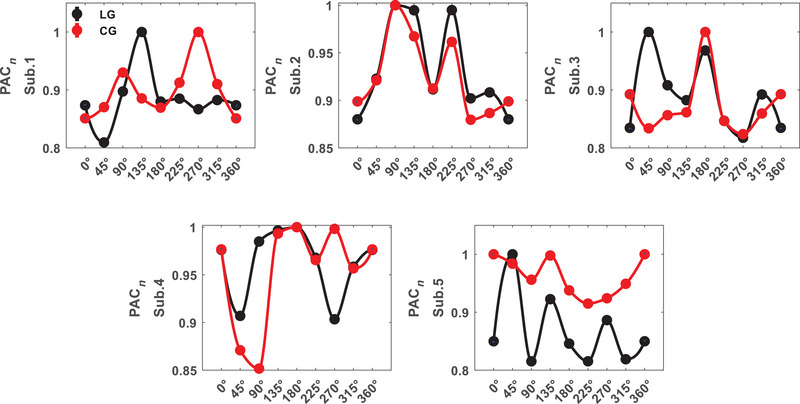
Phase–amplitude coupling (PAC) tuning curve for area V1 (PAC*_n_*: normalized to max(PAC)).

We investigated which pairs of subbands result in significant differences between orientations in area V1 by ANOVA test with within‐subject factor stimulus. It is observed that *θ*–*H* subbands have meaningful differences for all the subjects (*p* = 0.01 ± 0.004) as shown in Figure 1. In the following, it is focused on the selective subbands *θ*–*H* to investigate PAC tuning curves for stimuli and their differences. Figure 2 shows the results for each subject (Sub.1–Sub.5). The PAC values depicted in the figure are normalized to the maximum values. Different trends are observed in the tuning curves for two types of stimuli that shows stimulus effective role on perception. The behavioral study represented the perception of first‐order stimulus (LG). Euclidean distance was used to evaluate the similarity between first‐ and second‐order stimulus responses (it was calculated for 30 trials of each orientation and then averaged over orientations). This measure for area V1 subjects is $d_{\mathrm{PA}C_{1}}$ = 0.012, $d_{\mathrm{PA}C_{2}}$ = 0.003, $d_{\mathrm{PA}C_{3}}$ = 0.009, $d_{\mathrm{PA}C_{4}}$= 0.008, and $d_{\mathrm{PA}C_{5}}$= 0.009 ($d_{\mathrm{PA}C_{V1}}$ = 0.008 ± 0.003). We also considered stimulus type as the difference factor that in several subbands have meaningful differences for all the subjects, including *δ*–*H* (*p* = 2.4 × 10^−3^ ± 1.4 × 10^−3^). These results indicate correlation between phase of lower than 8 Hz band signal and amplitude of above 100 Hz band signal can be significantly different for orientations and stimulus conditions simultaneously.

**FIGURE 3:**
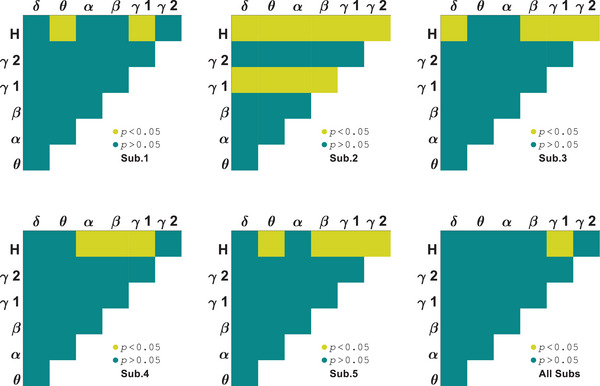
Subbands orientation selectivity of area LM by analysis of variance (ANOVA) test on phase–amplitude coupling (PAC).

**FIGURE 4:**
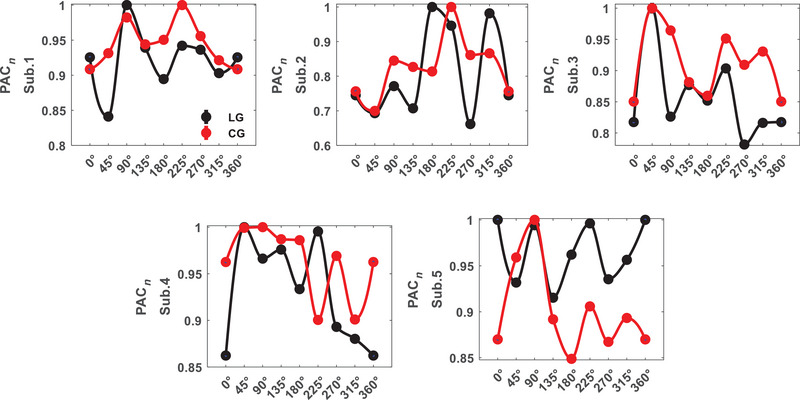
Phase–amplitude coupling (PAC) tuning curve for area LM (PAC*_n_*: normalized to max(PAC)).

The two‐way ANOVA test on area LM PAC showed the selectivity of several subbands for each subject. As illustrated in Figure 3, there are significant differences in pairs of multiple subbands and *H*‐band, but the PAC observed between *γ*1–*H* subbands shows significant differences among all subjects (*p* = 0.03 ± 0.006). There is no difference in the couplings between amplitude and phase of below 30 Hz frequency bands. We focused on this pair of subbands (*γ*1–*H*) for tuning curves investigations which are shown in Figure 4. The PAC values depicted in the figure are normalized to the maximum values. Euclidean distance for area LM subjects are $d_{\mathrm{PA}C_{1}}$ = 0.016, $d_{\mathrm{PA}C_{2}}$ = 0.021, $d_{\mathrm{PA}C_{3}}$ = 0.009, $d_{\mathrm{PA}C_{4}}$ = 0.008, and $d_{\mathrm{PA}C_{5}}$ = 0.008 ($d_{\mathrm{PA}C_{\mathrm{LM}}}$= 0.012 ± 0.006). The distance has increased in comparison to area V1. There is no difference in the couplings of *γ*1‐band phase and *H*‐band amplitude under stimulus conditions. However, a significant difference was found in the PAC of *δ* and *γ*1 subbands for all subjects (*p* = 3.7 × 10^−4^ ± 3.4 × 10^−4^). These findings suggest that PAC measure in area LM is not functional for both orientation and stimuli type together.

**FIGURE 5:**
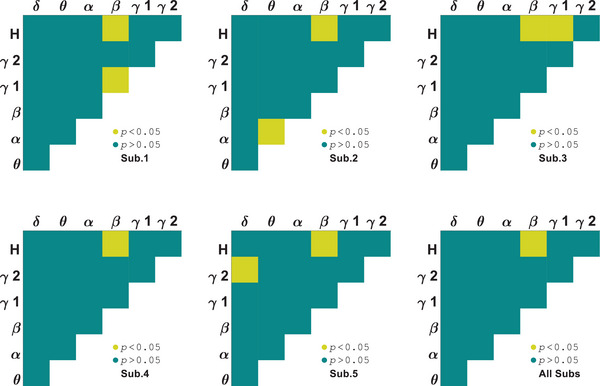
Subbands orientation selectivity of area V1 by analysis of variance (ANOVA) test on amplitude–amplitude coupling (AAC).

**FIGURE 6:**
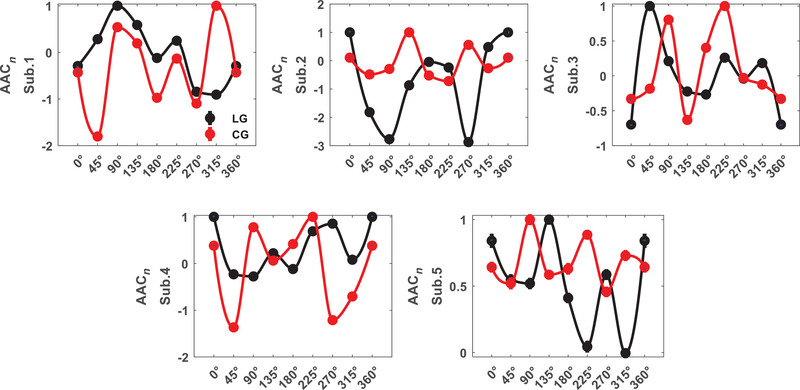
Amplitude–amplitude coupling (AAC) tuning curve for area V1 (AAC*_n_*: normalized to max(AAC)).

Another measure used to investigate the selectivity of areas V1 and LM to orientations and stimuli was AAC. A two‐way ANOVA test was used to identify orientation selective subbands for each subject. Amplitude coupling of signals below 12 Hz with themselves and those above 30 Hz did not have any orientation related discriminant. As seen in Figure 5, the correlation between amplitudes of *β* and *H* subbands is orientation selective for all of the area V1 subjects (*p* = 0.007 ± 0.002). In contrast to PAC, area V1 has fewer selective pair bands in AAC. Figure 6 illustrates that both stimuli have similar and closely matched tuning curves for each subject. The AAC values depicted in the figure are normalized to the maximum values. Euclidean distance for area V1 subjects are $d_{\mathrm{AA}C_{1}}$ = 0.101, $d_{\mathrm{AA}C_{2}}$ = 0.133, $d_{\mathrm{AA}C_{3}}$= 0.086, $d_{\mathrm{AA}C_{4}}$ = 0.072, and $d_{\mathrm{AA}C_{5}}$ = 0.106 ($d_{\mathrm{AA}C_{V1}}$= 0.100 ± 0.020). In the comparison of stimulus type, several subbands showed significant differences including *β*–*γ*2 that were subbands close to our selected one (*p* = 0.003 ± 0.001). These results indicate amplitude correlation between 12 and 30 Hz and more than 70 Hz subbands can be significantly differenced for orientations and stimuli together in area V1.

**FIGURE 7:**
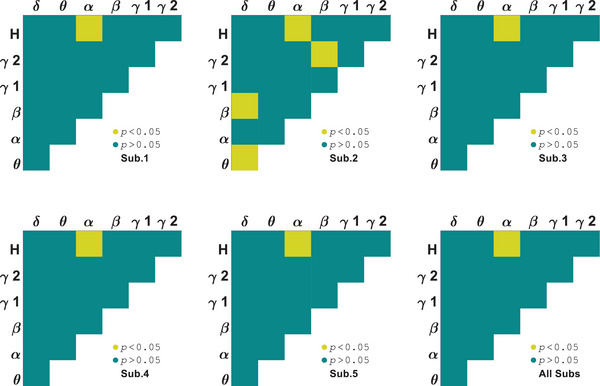
Subbands orientation selectivity of area LM by analysis of variance (ANOVA) test on amplitude–amplitude coupling (AAC).

**FIGURE 8:**
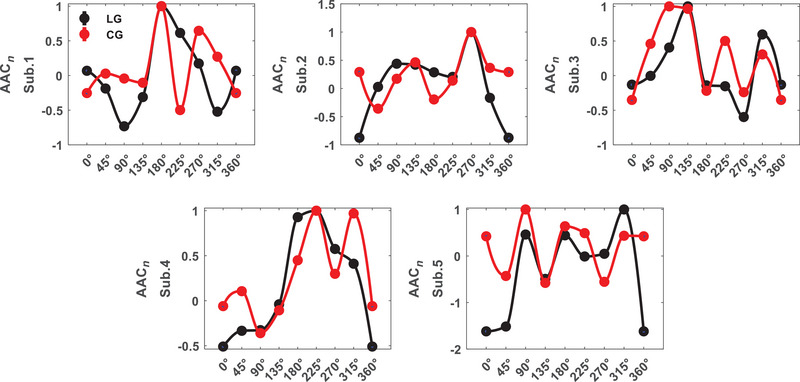
Area LM orientation tuning by amplitude–amplitude coupling (AAC; AAC*_n_*: normalized to max(AAC)).

Area LM amplitude coupling exhibits *α*–*H* selectivity to orientations for all the subjects (*p* = 0.007 ± 0.003). As seen in Figure 7, all subjects, except subject 2, showed a difference only in *α*–*H* coupling. Tuning curves of couplings between α and H bands amplitudes shown in Figure 8. Euclidean distance for area LM subjects are $d_{\mathrm{AA}C_{1}}$ = 0.130, $d_{\mathrm{AA}C_{2}}$ = 0.156, $d_{\mathrm{AA}C_{3}}$ = 0.075, $d_{\mathrm{AA}C_{4}}$ = 0.069, and $d_{\mathrm{AA}C_{5}}$ = 0.118 ($d_{\mathrm{AA}C_{\mathrm{LM}}}$ = 0.11 ± 0.037). The distance has increased in comparison to area V1. For each subject, there were selective subbands to stimulus type but *β*–*H* coupling was selective to stimulus type for all the subjects (*p* = 0.007 ± 0.004). These results indicate that amplitudes in the 8–30 Hz and more than 100 Hz subbands may be selective to both stimulus type and orientation.

## 4 Discussion

Pursuant to preceding studies on the nonhuman primate and rodent visual cortex, orientation selectivity of a visual stimulus is an importunate property of neurons in V1 (Priebe 2016; Dubey and Ray 2020; Long et al. 2015). The previous study (Khastkhodaei et al. 2016) on the spike level in both areas V1 and LM showed that responsive neurons to CG stimuli exhibit generally weaker and less selective tuning compared to LG stimuli. Some studies have shown the importance of mesoscale‐level analysis in revealing brain processing while visual perception (Henrie and Shapley 2005; Berens 2008; Berens et al. 2008; Jia et al. 2011; Gail et al. 2004). We were encouraged to assess brain circuitry during visual passive perception, with CFC analysis.

We used the recorded neural data in V1 and LM of 10 mice, five mice for each area. Recording sessions from each area were separated. Thirty‐two‐channel silicon probes configured in four shanks were used, but due to the proximity of the channels on each shank, their averages were used. In the experiment, the mice performed a passive visual task, where a drifting sinusoidal grating with different orientations was displayed as the stimulus. There were two types of stimulation, LG and CG. After decomposing the signal into seven frequency bands and extracting trials, PAC and AAC of the LFPs were extracted to assess the orientation discrimination during visual perception.

Our results showed that AAC can be a valuable measure in both areas (V1 and LM) that could reveal different mechanisms of neural responses for orientations and stimuli conditions. For orientation responses, this disclosure was observed in the amplitude coupling between beta band signal and above 100 Hz band signal for area V1, and between alpha band signal and above 100 Hz band signal for area LM. This result was true for all the subjects. Considering the significant bands for the type of stimuli highlights the importance of higher than 70 Hz and lower than 30 Hz frequency bands in modulations with perception.

For all the subjects, there was discriminative PAC to orientations in V1 and LM areas. This significant performance was observed in the coupling between the phase of lower than 8 Hz band signal and amplitude of above 100 Hz band signal for area V1 for both orientations and stimuli conditions. However, in area LM subjects, we observed that the coupling between the phase of gamma1 band signal and the amplitude of above 100 Hz band signal differed significantly depending on the orientation. On the other hand, separable coupling for stimuli conditions was observed between the phase of delta band signal and the amplitude of gamma1 band signal for all the subjects. These results indicate that PAC plays an important role in the manifestation of perception mechanisms in area V1 and LM.

**FIGURE 9:**
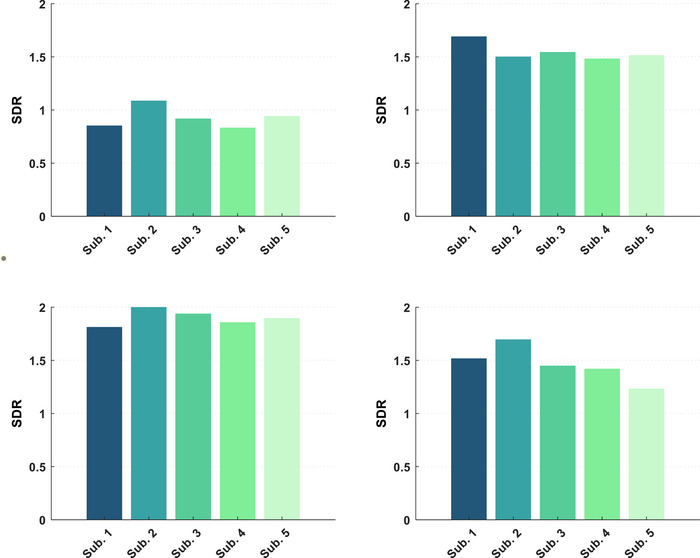
Standard deviation ratio (SDR) for each subject. Right column: area LM, left column: area V1, upper row: amplitude–amplitude coupling (AAC), lower row: phase–amplitude coupling (PAC).

The standard deviations ratio (SDR) of two types of stimuli is considered as a selectivity measure of area (SDR = SD_LG_/SD_CG_). On average, SDR for amplitudes coupling in area V1 is 0.91, while for area LM it is 1.54. Area LM subjects had ratios greater than 1, indicating different variations in AAC tuning between two stimuli conditions and reducing the range of activity for the second‐order stimulus. But in area V1, the ratios were almost 1, indicating similar variations in AAC tuning between two stimuli conditions. The average SDR for PAC is 1.90 in area V1 subjects and 1.45 in area LM subjects that represent this type coupling diminution of the areas for second‐order stimuli. Figure 9 displays the SDR value of each subject for two different areas and coupling types.

In the comparison of two areas, AAC values in area V1 are greater than area LM ($m_{V1,\;\mathrm{LG}}=3.36\;\times \;10^{-2},\;\;\;m_{V1,\;\mathrm{CG}}=\;\;3.14\;\times \;10^{-2},\;\;m_{\mathrm{LM},\;\mathrm{LG}}=1.85\;\times \;10^{-2},\;\;\mathrm{and}\;\;m_{\mathrm{LM},\;\mathrm{CG}}=2.95\;\times \;10^{-2}$; means are over all the subjects and orientations) and PAC value in area LM are greater than area V1 ($m_{V1,\;\mathrm{LG}}=4.61\;\times \;10^{-2}$, $m_{V1,\;\mathrm{CG}}=4.54\;\times \;10^{-2}$, $\;m_{\mathrm{LM},\;\mathrm{LG}}=5.62\;\times \;10^{-2}$, $\;m_{\mathrm{LM},\;\mathrm{CG}}=5.1\;\times \;10^{-2}$). Based on the aforementioned drops and SDR values, it can be concluded that PAC is more effective in area V1 for both orientation and stimulus condition. On the other hand, area LM shows selectivity in terms of both orientation and stimulus conditions in its AAC.

## 5 Conclusions

In conclusion, the present study investigated the functional role of CFCs in the perception of first‐ and second‐order visual stimuli. Our findings revealed important insights in this area. The results suggest that neural correlates of alpha–beta (8–30 Hz) and high‐band (100–200 Hz) oscillations in area LM, and correlates of delta–theta (1–8 Hz) and high‐band (100–200 Hz) oscillations in area V1 can be effectively selective to the grating orientations, which opens new horizons for further studies explaining the nature of orientation selectivity. In both areas, the significant role of high‐band and low‐band oscillations of LFPs discloses the reliability of these bands in visual perception. Hence, the significant role of LFP signals has been demonstrated in mice visual perception.

## Author Contributions

**Zahra Ebrahimvand**: methodology, software, writing–original draft, writing–review and editing. **Mohammad Reza Daliri**: conceptualization, methodology, supervision, writing–review and editing.

## Ethics Statement

All procedures complied with the European Communities Council Directive 2010/63/EC and the German Law for Protection of Animals, and were approved by local authorities, following appropriate ethics review (Khastkhodaei et al. 2016).

## Conflicts of Interest

The authors declare no conflicts of interest.

### Peer Review

The peer review history for this article is available at https://publons.com/publon/10.1002/brb3.70360

## Data Availability

The data that support the findings of this study are available from the corresponding author upon reasonable request. The full description of the dataset used for this study can be found in Khastkhodaei et al. (2016).
